# Changes in the incidence of acute bacterial meningitis caused by *Streptococcus pneumoniae* and the implications of serotype replacement in children in Colombia after mass vaccination with PCV10

**DOI:** 10.3389/fped.2022.1006887

**Published:** 2022-09-23

**Authors:** Juan David Farfán-Albarracín, Germán Camacho-Moreno, Aura Lucia Leal, Jaime Patiño, Wilfrido Coronell, Iván Felipe Gutiérrez, Sandra Beltrán, Martha I. Álvarez-Olmos, Cristina Mariño, Rocio Barrero, Juan Pablo Rojas, Fabio Espinosa, Catalina Arango-Ferreira, Maria Alejandra Suarez, Monica Trujillo, Eduardo López-Medina, Pio López, Hernando Pinzón, Nicolás Ramos, Vivian Marcela Moreno, Anita Montañez

**Affiliations:** ^1^Red Neumocolombia, Bogotá, Colombia; ^2^Departamento de Pediatría, Facultad de Medicina, Universidad Nacional de Colombia, Bogotá, Colombia; ^3^HOMI-Fundación Hospital Pediátrico La Misericordia, Bogotá, Colombia; ^4^Hospital Infantil Universitario de San José, Bogotá, Colombia; ^5^Departamento de Microbiología, Facultad de Medicina, Universidad Nacional de Colombia, Bogotá, Colombia; ^6^Grupo para el Control de la Resistencia Bacteriana en Bogotá (GREBO), Bogotá, Colombia; ^7^Fundación Valle de Lili, Cali, Colombia; ^8^Hospital Infantil Napoleón Franco Pareja, Cartagena, Colombia; ^9^Clínica Infantil Colsubsidio, Bogotá, Colombia; ^10^Clínica Infantil Santa María del Lago-Colsánitas, Bogotá, Colombia; ^11^Clínica Universitaria Colombia-Clínica Pediátrica Colsanitas, Bogotá, Colombia; ^12^Fundación Cardioinfantil-Instituto de Cardiología, Bogotá, Colombia; ^13^Hospital Militar Central, Bogotá, Colombia; ^14^Hospital Universitario Clínica San Rafael, Bogotá, Colombia; ^15^Unidad de Servicios de Salud Santa Clara, Subred Centro Oriente, Bogotá, Colombia; ^16^Fundación Clínica Infantil Club Noel, Cali, Colombia; ^17^Facultad de Ciencias de la Salud, Universidad Libre Seccional Cali, Cali, Colombia; ^18^Facultad de Salud, Universidad del Valle, Cali, Colombia; ^19^Hospital Universitario San Vicente Fundación, Medellín, Colombia; ^20^Departamento de Pediatría, Facultad de Medicina, Universidad de Antioquia, Medellin, Colombia; ^21^Unidad de Servicio de Salud Tunal, Bogotá, Colombia; ^22^Hospital Pablo Tobón Uribe, Medellín, Colombia; ^23^Centro Médico Imbanaco, Cali, Colombia; ^24^Hospital Universitario del Valle, Cali, Colombia; ^25^Los COBOS Medical Center, Bogotá, Colombia

**Keywords:** pneumococcal meningitis, pediatrics, infections, vaccines, microbiology, serotype

## Abstract

**Introduction:**

Acute bacterial meningitis (ABM) is a public health problem. The disease has reemerged after the introduction of pneumococcal conjugate vaccines (PCVs) due to an increase in serotypes that are not covered. The objective was to determine the changes in the disease incidence before and after the introduction of the 10-valent vaccine (PCV10) in Colombia.

**Methods:**

This multicenter study was conducted in 17 hospitals in Colombia. Data were collected from January 2008 to December 2019 in 10 hospitals in Bogotá and from January 2017 to December 2019 in seven hospitals in Cali, Medellín and Cartagena. The data were grouped into three periods: 2008–2011, 2012–2015, and 2016-2019.

**Results:**

Of the 706 cases of invasive pneumococcal disease, 81 (11.4%) corresponded to meningitis. The relative incidence in Bogotá in the first period was 0.6 per 100,000 patients ≤ 5 years, decreased to 0.4 per 100,000 patients ≤ 5 years in the second period and increased in the third period to 0.7 per 100,000 patients ≤ 5 years. Serotypes covered by PCV10 decreased from 75 to 9.1%, with Spn19A (31.8%) and Spn34 (13.6%) emerging in the third period. Increased resistance to penicillin (13 to 37%) and to ceftriaxone (5.9 to 16%) was due to the emergence of multidrug-resistant Spn19A. The total mortality rate was 23.5% and increased from 12 to 33%.

**Conclusions:**

ABM due to pneumococcus has high morbidity and mortality rates. Reemergence of the disease has been observed due to the inclusion of polymerase chain reaction (PCR) for diagnosis and replacement of circulating serotypes after the introduction of PCV10, with an increase in Spn19A, which causes death and exhibits antimicrobial resistance. Continued surveillance is needed.

## Introduction

Acute bacterial meningitis (ABM) is a public health problem with high morbidity and mortality rates, especially in children under 5 years of age. One of the most common causative microorganisms is *Streptococcus pneumoniae* (Spn). In 2015, Wahl et al. estimated 83,900 cases of ABM caused by pneumococcus in children under 5 years of age worldwide, with an incidence of 13 in 100,000 and a mortality rate of 44%. Of the 37,900 deaths, 53.8% occurred in Africa and 26.9% in Southeast Asia. In America, 2,300 cases of ABM due to pneumococcus were reported in children under 5 years of age (incidence of 3 in 100,000), and 600 (27%) children died (1). In Colombia, in 2019, 64 cases of pneumococcal meningitis were reported in children under 18 years of age, and 35 occurred in children under 5 years of age (incidence 1.03 cases per 100,000 children under 5 years of age), with a mortality rate of 18%. In this age group, mortality rates as high as 44% were reported in 2017 (2, 3).

The World Health Organization (WHO) established a roadmap to defeat meningitis by 2030. Pillar 2 refers to optimizing diagnosis and treatment, for which molecular diagnostic techniques have been implemented that have allowed researchers to improve microorganism detection, and pillar 3 refers to the need to determine the epidemiology of this disease, strengthening surveillance systems and encouraging research on the subject (4). Vaccines against *Streptococcus pneumoniae, Neisseria meningitidis* and *Haemophilus influenzae* type b have been developed to control this disease (4, 5). Worldwide, a decrease in the incidence of pneumococcal meningitis has been observed after the application of pneumococcal conjugate vaccines (PCVs). In recent years, serotypes not included in PCVs that are applied systematically have emerged, leading to a reemergence of the disease (6).

The main clinical manifestations are fever, headache, nuchal rigidity, confusion, an altered state of consciousness, general malaise, epileptic seizures and vomiting, depending mainly on the age of the patient (7). Other nonspecific symptoms have been described as clinical characteristics in children, including hypothermia, rejection of the oral administration route, bulging of fontanelles, irritability and alternation between irritability and drowsiness (5, 8). In addition, in children under 12 months of age, meningeal signs may be absent, further complicating the diagnostic process (5, 9, 10).

One factor that has changed the behavior of the disease worldwide is vaccination (11). For *S. pneumoniae*, conjugate vaccines have been designed against seven serotypes (4, 6B, 9V, 14, 18C, 19F, 23F), 10 serotypes (PCV 7 + 1, 5, 7F), 13 serotypes (PCV 10 + 3, 6A, 19A), 15 serotypes (PCV 13 + 22F, 33F) and 20 serotypes (PCV 15 + 8, 10A, 11A, 12F, 15B). As of June 2019, 37 countries in the Americas had introduced some of the PCVs into their schedule (12). In Colombia, the first vaccine introduced was the seven-valent vaccine (PCV7), initially in the city of Bogotá in 2008, and subsequently, the 10-valent vaccine (PCV10) was introduced nationwide in 2012.

The *Neumocolombia* network is an initiative of the Central Chapter of the Colombian Association of Infectious Diseases (Asociación Colombiana de Infectología–ACIN) and the Colombian Society of Pediatrics (Sociedad Colombiana de Pediatría-SCP) that monitors the behavior of invasive pneumococcal disease (IPD). The objective of this study was to determine the clinical, microbiological and epidemiological changes that have occurred in pneumococcal meningitis in children before and after the introduction of mass vaccination in Colombia.

## Methodology

Patients aged younger than 18 years with ABM confirmed to be caused by *S. pneumoniae* were included and were defined as patients with a suggestive clinical presentation, cerebrospinal fluid (CSF) findings compatible with ABM and isolation of the microorganism in blood culture and/or CSF culture and/or detection by polymerase chain reaction (PCR) in CSF. Data were collected ambispectively from January 1, 2008, to December 31, 2019, in the city of Bogotá DC and from January 1, 2017, to December 31, 2019, in Cartagena, Cali and Medellín, for a total of 17 centers in Colombia. A questionnaire was designed for online data collection.

For bacterial identification and susceptibility, automatized methods were used (16 centers–Vitek 2™, and one center–BD Phoenix™ technology). The CLSI 2018 criteria for minimum inhibitory concentrations (MICs) were applied to classify susceptible, intermediate and resistant isolates for each antibiotic drug. As part of routine surveillance, isolates should be sent to local health departments (Secretarías Departamentales de Salud–SDS), where the isolates are reconfirmed and sent to the National Institute of Health (INS), where serotyping by the Quellung method and by polymerase chain reaction, as appropriate, is performed. The serotyping data were taken from the report sent by the SDS and INS to the institutions.

The data were grouped according to the start of mass vaccination with PCV in Colombia in 2012 into three different periods: prevaccination period from 2008 to 2011, transition period from 2012 to 2015 and postvaccination period from 2016 to 2019. Subsequently, the data were analyzed with the R statistics 4.1.1 tool (R Foundation for Statistical Computing, Vienna, Austria) *via* the software package RStudio version 1.4.1717 (RStudio, PBC, Boston, MA), initially with descriptive methods, including means, medians, standard deviations and interquartile ranges, according to the characteristics of the different variables. The values of different variables were compared, such as cell counts in blood and CSF and levels of glucose and C-reactive protein, according to specific outcomes, which included mortality and the presence of complications, using Wilcoxon rank tests or *T*-tests according to the characteristics of the evaluated variable. Subsequently, reference points were established for the significant variables after considering the clinical relevance of each variable. For categorical variables, the chi-square test or Fisher's exact test was used, according to the characteristics of the population, and the ORs were calculated for different outcomes, as previously mentioned.

## Results

During the study period, 706 cases of IPD were identified. Of these, 81 cases (11.4%) corresponded to meningitis, 66 (81.4%) cases were from Bogotá D.C., and 15 (18.5%) cases were from outside Bogotá D.C. (seven from Cali, five from Medellín and three from Cartagena). A total of 17.3% of patients had concomitant pneumonia. The median age was 39 months (range 0–211 months), 61.7% of patients were male, 66.6% of patients were affiliated with the contributory regimen, and 29.6% were affiliated with the subsidized regimen. In 69.1% of patients, the admission diagnosis was neuroinfection, followed by pneumonia (4.9%), febrile syndrome (2.4%) and complex febrile seizures (2.4%). Table 1 shows the demographic data and characteristics of the population.

**Table 1:** Demographic and clinical characteristics of patients with pneumococcal meningitis.

**Variable**	**Bogotá D.C. *n* = 66 (%)**	**Outside Bogotá D.C. *n* = 15 (%)**	**Total *n* = 81 (%)**
**Age in months (median, range)**	34.5 (0–211)	64 (2–149)	39 (0–211)
**Age groups**			
0–3 months	12 (18.2)	3 (20)	15 (18.5)
4–12 months	15 (22.7)	2 (13.3)	17 (21)
13–35 months	7 (10.6)	1 (6.7)	8 (9.9)
36–59 months	4 (6.1)	1 (6.7)	5 (6.2)
≥ 60 months	28 (42.4)	8 (53.3)	36 (44.4)
**Sex**			
Male	43 (65.2)	7 (46.7)	50 (61.7)
Female	23 (34.8)	8 (53.3)	31 (38.3)
**ICU requirement**	48 (72.7)	12 (80)	60 (74)
**Mortality**	15 (22.7)	4 (26.7)	19 (23.5)
**Complications**			
Shock	29 (43.9)	8 (53.3)	37 (45.7)
Ventilatory failure	31 (47)	6 (40)	37 (45.7)
Coma	21 (31.8)	0 (0)	21 (25.9)
Status epilepticus	16 (24.2)	2 (13.3)	18 (22.2)
Vasculitis/stroke	9 (13.6)	1 (6.7)	10 (12.3)
Hygromas	6 (9.1)	0 (0)	6 (7.4)
Subdural empyemas	5 (7.6)	0 (0)	5 (6.2)
Hydrocephalus	4 (6.1)	0 (0)	4 (4.9)
Cerebritis	2 (3)	1 (6.7)	3 (3.7)
CVST	3 (4.5)	0 (0)	3 (3.7)
Ventriculitis	2 (3)	1 (6.7)	3 (3.7)
Brain abscess	0 (0)	2 (13.3)	2 (2.5)
**Vaccination data were obtained**	43 (65.2)	13 (86.7)	56 (69.1)
**PCV7 conjugate vaccine**	2 (3)	0 (0)	2 (2.4)
One dose	0 (0)	0 (0)	0 (0)
Two doses	1 (1.5)	0 (0)	1 (1.2)
Three doses	1 (1.5)	0 (0)	1 (1.2)
**PCV10 conjugate vaccine**	17 (25.8)	10 (66.7)	27 (33.3)
One dose	6 (9.1)	2 (13.3)	8 (17.9)
Two doses	6 (9.1)	2 (13.3)	8 (17.9)
Three doses	5 (7.6)	4 (26.7)	9 (16.1)
Four doses	0 (0)	2 (13.3)	2 (3.6)
**PCV13 conjugate vaccine**	0 (0)	1 (6.7)	1 (1.2)
One dose	0 (0)	0 (0)	0 (0)
Two doses	0 (0)	1 (6.7)	1 (1.2)
Three doses	0 (0)	0 (0)	0 (0)
**No data on the vaccine type**	1 (1.5)	0 (0)	1 (1.2)
**Polysaccharide vaccine**	0 (0)	2 (13.3)	2 (3.6)
After PCV10	0 (0)	1 (6.7)	1 (1.2)
After PCV13	0 (0)	1 (6.7)	1 (1.2)
**Not vaccinated**	20 (30.3)	5 (33.3)	25 (30.9)
**No vaccination data**	23 (34.8)	2 (13.3)	25 (30.9)
**Vaccination periods**			
Prevaccination period	25 (37.9)	0 (0)	25 (30.9)
Transition period	17 (25.8)	0 (0)	17 (21)
Postvaccination period	24 (36.4)	15 (100)	39 (48.2)

PCV, pneumococcal conjugate vaccine; CVST, cerebral venous sinus thrombosis; ICU, intensive care unit.

Of the total patients with meningitis, 45 (55.5%) were younger than 5 years of age, and 32 (39.5%) corresponded to the population 12 months old or younger (Table 1). Sixteen percent of patients used an antibiotic prior to the diagnosis of meningitis; in most cases, amoxicillin or ceftriaxone was used. The most frequent antecedent was CSF fistula in 17.3% of patients, followed by immunodeficiency and chronic neurological disease in 3.7%, chronic pneumopathy and craniofacial malformations in 2.5% and kidney failure and chronic endocrinological disease in 1.2% each. Vaccination data were obtained for 56 (69.1%) of the patients, 25 of whom (30.9%) had not received any vaccine, while 27 (33.3%) had received at least one dose of PCV10. Eleven of these patients (19.7%) had received a complete vaccination schedule, two patients had received PCV7, one received PCV13, and two received PSP23 after PCV10 and PCV13 (Table 1). Eighty percent and 100% of patients who were infected with Spn19A and Spn34, respectively, had received at least one dose of PCV10 (Supplementary material, Supplementary Figure S2).

The most common symptoms were fever, hyporexia, drowsiness and vomiting. Epileptic seizures ranked fifth, with the rate reaching 48.2%. Specific symptoms of acute meningitis, such as headache, nuchal rigidity or signs of meningeal irritation, were present in 39.5, 34.6, and 14.8% of patients, respectively. The frequency of these symptoms differed according to the age group of the patient (Supplementary material, Supplementary Figure S1). A higher prevalence of epileptic seizures was observed in patients younger than 12 months; in contrast, classic signs of acute meningitis, such as those described above, were present at a higher prevalence in children older than 36 months. Patients with Spn19A infections showed peaks between 13 and 36 months of age (44.4% of cases) after the last dose of the vaccine scheme, and their symptoms were similar to those previously described for this group, with a predominance of fever (88.9%), followed by irritability (66.7%), vomiting (44.4%), drowsiness (44.4%) and poor feeding (44.4%). The median symptom duration on admission was 3 days. The most prominent findings in the CSF studies were hypoglycorrhachia, with a median of 10 mg/dl [interquartile range (IQR) 1.6–39.5 mg/dl]; a low glycorrhachia/serum glucose ratio, with a median of 0.05 (IQR 0.01–0.29); hyperproteinorrhea, with a median of 272.1 (IQR 115.2–335.4); pleocytosis, with a median of 220 (IQR 36–1250) and a polymorphonuclear predominance; and positive Gram staining for gram-positive cocci in 70.4% of patients (Supplementary material, Supplementary Table S1).

Neuroimaging was performed in 54 (66.7%) patients with the cranial tomography (CT) scanning modality and in 24 (29.6%) patients with the brain magnetic resonance imaging (MRI) modality. The most frequent findings were parameningeal foci (mastoiditis or sinusitis), followed by normal neuroimaging (31.5 and 30.9% on CT and 37.5 and 29.2% on MRI, respectively). Cerebral edema was the third most frequent finding on tomography scans (27.8%). Likewise, meningeal enhancement was observed on brain MRI in 25% of patients, while this finding was observed on tomography scans in 5.6% of patients. These findings were more common in the Spn19A infection group, with parameningeal foci (CT: 60%, MRI: 50%), cerebral edema (CT: 20%, MRI: 50%), meningeal enhancement (CT: 20%, MRI: 50%) and normal images (CT: 20%, MRI: 50%) noted in a limited sample of patients (*n* = 5 for CT and *n* = 2 for MRI).

**Figure 1:**
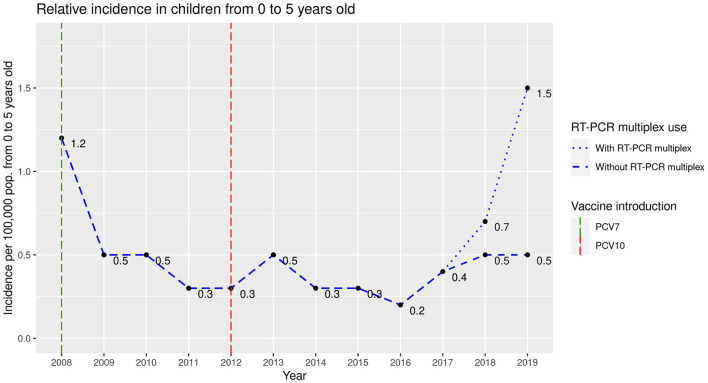
Relative incidence of acute bacterial meningitis in children between 0 and 5 years of age in Bogotá.

Relative incidence data were calculated with respect to the population aged 0 to 5 years in Bogota, including data from 10 hospitals in this city with data available from the beginning of the study. As shown in Figure 1, an increase in the number of cases was observed in the postvaccination period (2016 to 2019), with an average incidence of 0.69 cases per 100,000 inhabitants under 5 years of age, compared with the data from the transition period (2012 to 2015), which showed an average incidence of 0.35 cases per 100,000 inhabitants under 5 years of age, and the prevaccination period, where the average incidence was 0.62 cases per 100,000 inhabitants. In 75 (92.6%) patients, *S. pneumoniae* was isolated by CSF culture or blood culture, while in six patients (7.4%), it was performed only by molecular tests [real-time PCR (RT–PCR) multiplex-FilmArray^®^] for one patient in 2018 and five in 2019. This technology was not available in the first two periods of the study, and thus, the incidence in the postvaccination period (2016 to 2019) was also calculated without including these cases at 0.4 cases in 100,000 children under 5 years of age, which is lower than the incidence in the prevaccination period and higher than the incidence in the transition period (Figure 1). The data from the network represent 47% of the cases of IPD that Bogotá reports to the surveillance system (13).

**Figure 2:**
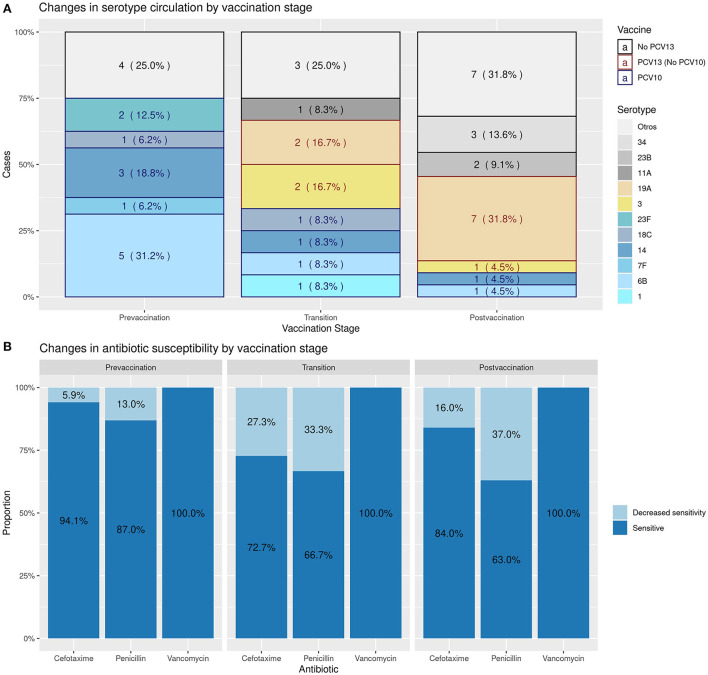
Changes in the circulation of serotypes and in bacterial resistance according to the vaccination period.

The serotype was obtained in 50 (61.7%) cases; the bacterial serotype was not obtained in any of the six cases detected using RT–PCR. When performing the analysis of the serotypes, the serotypes included in PCV10 were rare in vaccinated patients, while serotypes 19A and 34 were more frequent in vaccinated patients than in unvaccinated patients (Supplementary material, Supplementary Figure S2). When the serotypes were analyzed according to the vaccination period, the serotypes included in the PCV10 vaccine decreased in prevalence from 75 to 9.1% in the prevaccination and postvaccination periods, respectively, while the emerging serotypes were mainly represented by Spn19A and Spn34, with 31.8 and 13.6% prevalence, respectively, in the postvaccination period (Figure 2).

Likewise, Figure 2 shows the antimicrobial sensitivity to the most frequently used antibiotics to treat IPD, such as penicillin, cefotaxime and vancomycin, throughout the vaccination period. The sensitivity to the first two drugs decreased progressively, with a decreased sensitivity (intermediate or resistant) of 40% for penicillin and 16% for third-generation cephalosporins in the postvaccination period. Specifically, Spn19A's resistance increased from none in the transition period to 66.7% in the postvaccination period for both penicillin and cefotaxime. All isolates were sensitive to vancomycin.

The most empirically used antibiotics were third-generation cephalosporins (90.1%), followed by vancomycin (69.1%) and ampicillin (9.9%). The empirical use of glycopeptides increased from 60% in the prevaccination period to 71.8% in the postvaccination period. Likewise, vancomycin was maintained as part of the treatment after the results of the susceptibility testing in 0% of the patients in the prevaccination period, in 11.8% in the transition period and in 12.8% in the postvaccination period.

The rate of dexamethasone use in combination with antibiotics was 22.2%; dexamethasone was started simultaneously with antibiotics in 50% of patients and started before antibiotics in 22.2%. The median hospital stay duration was 16 days, and 60 (74%) patients required admission to the intensive care unit, with a median stay of 4 days.

The most frequent complications were severe shock, ventilatory failure, coma, status epilepticus and central nervous system vasculitis or stroke (Table 1). Some important differences were observed between the no Spn19A and Spn19A groups in severe shock (46.3 vs. 55.6%), cerebritis (2.4 vs. 11.1%) and status epilepticus (26.8 vs. 11.1%), none of which were statistically significant. The mortality rate of the disease reached 23% and varied, with an important increase since the prevaccination period from 12% compared with those in the transition (17.6%) and postvaccination (33.3%) periods. This difference between the prevaccination period and postvaccination period was not statistically significant (*p* = 0.104).

In the bivariate analysis, associations were observed between lower blood counts of leukocytes, neutrophils and monocytes, higher C-reactive protein levels, lower glucose levels in CSF and a younger age with a greater probability of presenting some complication. Finally, lower leukocyte and neutrophil counts, lower hemoglobin levels, a higher C-reactive protein level in blood and a lower glucose level in CSF were associated with higher mortality (Table 2).

**Table 2:** Variables associated with greater complications and greater lethality.

**Outcome (Oc)**	**Present**	**Absent**	**OR (95% CI)**	***p***
**Complications (any)**				
Age ≤ 24 months (*n* = 77)	27	9	3.7 (1.4–10.4)	0.006
Glucose level in CSF ≤ 20 mg/dl (*n* = 67)	27	13	4.0 (1.4–12.0)	0.006
CRP level ≥ 100 mg/l (*n* = 38)	15	3	8.4 (1.9–49)	0.004
Leukocyte count < 11,000/mm^3^ (*n* = 72)	17	4	4.2 (1.3–16.8)	0.017
Neutrophil count < 7500/mm^3^ (*n* = 72)	19	5	4.0 (1.3–13.9)	0.011
Monocyte count < 850/mm^3^ (*n* = 61)	27	12	4.6 (1.5–15.3)	0.004
Use of corticosteroids (*n* = 77)	8	9	1.8 (0.6–5.5)	0.28
Diagnosis different from neuroinfection upon admission (*n* = 77)	14	10	1.0 (0.4–2.7)	0.99
Serotype 19A (*n* = 50)	6	23	0.7 (0.1–4.0)	1
Resistance for penicillin (*n* = 63)	11	28	1.1 (0.3–3.3)	0.935
Decreased sensitivity for third-generation cephalosporine (*n* = 50)	6	23	0.4 (0.04–2.6)	0.441
**Mortality**				
Glucose level in CSF ≤ 20 mg/dl (*n* = 70)	13	30	9.8 (1.7–250.2)	0.007
Leukocyte count < 11,000/mm^3^ (*n* = 75)	10	13	4.1 (1.3–13.2)	0.008
Neutrophil count < 7,500/mm^3^ (*n* = 75)	10	16	3.1 (1.0–9.8)	0.047
Hemoglobin level ≤ 11 g/dl (*n* = 75)	11	14	4.7 (1.5–15.3)	0.004
CRP level ≥ 100 mg/l (*n* = 40)	12	8	23.4 (3.6–641.9)	< 0.000
Use of corticosteroids (*n* = 81)	4	14	1.1 (0.3–4.4)	1
Need for mechanical ventilation (*n* = 81)	16	21	9.7 (2.8–47.7)	< 0.000
Need for vasopressor/ionotropic support (*n* = 81)	16	16	14.2 (4.1–70.6)	< 0.000
Diagnosis of pneumonia (*n* = 81)	4	10	1.4 (0.3–5)	0.729
Diagnosis different from neuroinfection upon admission (*n* = 81)	7	18	1.4 (0.5–4.2)	0.519
Serotype 19A (*n* = 50)	4	10	0.4 (0.07–2.5)	0.245
Resistance for penicillin (*n* = 65)	4	12	1.2 (0.3–6.0)	1
Decreased sensitivity for third-generation cephalosporine (*n* = 53)	2	9	0.7 (0.1–8.9)	0.665

CRP, C-reactive protein; CSF, cerebrospinal fluid.

## Discussion

ABM due to *S. pneumoniae* in pediatric patients is a pathology with diverse clinical manifestations, making its diagnosis and timely treatment difficult. This study is one of the largest case series analyses of the disease, which exclusively includes the pediatric population. Likewise, all age pediatric groups are represented, with cases predominantly occurring in the first 36 months of life, including a slightly higher proportion of males, which has also been described in other cohorts (14–16).

In the present study, significant variation was observed in the most frequent symptoms according to the age group, with nonspecific manifestations observed in patients under 12 months of age. This diversity of symptoms, as well as their lack of specificity at early ages, has been reported in other local studies, where fever (93%), vomiting (78%), an altered state of consciousness (42%) and headache (40%) are described as the main symptoms (7). Symptoms did not significantly vary with Spn19A infection in this study. Likewise, symptoms similar to those mentioned in the present study were reported in a systematic review of the clinical characteristics of meningitis in children, and the best predictors of meningitis were bulging fontanelle, epileptic seizures and hyporexia, but the ideal combination of symptoms for diagnosis was unclear (17). Thus, a high diagnostic suspicion is necessary from admission to the emergency unit.

On the other hand, in terms of laboratory tests, hypoglycemia and hyperproteinorrhachia were observed, which are expected in the context of bacterial meningitis, with values similar to those reported in other studies (16). In previous studies, very high levels of C-reactive protein were associated with a higher rate of complications and sequelae of bacterial meningitis in children (18). However, in our study, C-reactive protein was also associated with mortality. In this study, blood leukocyte and neutrophil counts below the expected values, as well as the presence of anemia, were associated with mortality and complications. Other factors associated with mortality described in other studies, such as severe hypoglycemia (15), were also identified in our study.

Regarding the two neuroimaging techniques used, tomography and MRI, signs with high specificity for the diagnosis of meningitis are rare, their diagnostic value is scarce, and they do not replace biochemical and microbiological studies. The greatest utility of these studies is in detecting disease complications and not in the diagnostic process itself, as can be concluded from specific research on the subject (19, 20).

The trends in the incidence of the cases described (Figure 1), specifically in the city of Bogotá, correlated with the annual incidence among children under 5 years of age reported by the National Institute of Health in Colombia, with a rate of 1.19 per 100,000 children under 5 years of age in 2009 (21), which decreased to 0.51 per 100,000 children under 5 years of age in 2012 (22), remained stable between 0.54 and 0.63 per 100,000 children under 5 years of age between 2013 and 2018 (2, 23–27) and increased to 1.03 cases per 100,000 children under 5 years of age in 2019 (3). The implementation of molecular diagnostic techniques has increased the number of confirmed cases, allows the incidence and burden of the disease to be established more accurately and facilitates more timely treatment (28).

The behavior after mass vaccination reveals that in the first years after the introduction of vaccines, the incidence decreased, but a progressive increase in the relative incidence of ABM due to pneumococcus was observed in the postvaccination period, which may be mainly due to two factors: serotype replacement and the introduction of diagnostic strategies other than culture and/or antigen detection, such as detection using RT–PCR. Although the change in incidence without molecular testing was small, there was an increase respect to the minimum value (Figure 1). The phenomenon of serotype replacement has also been described in other regions of the world where PCVs have been introduced (14, 16, 29). The increase in the prevalence of serotypes not covered by PCV10 in IPD, especially Spn19A, has already been observed previously in Colombian cohorts of children and adults (30, 31), in Latin American cohorts (32) and in other regions of the world (6, 33). Notably, Spn34 has been reported only occasionally in patients with this disease.

On the other hand, we observed that resistance to third-generation cephalosporins increased from the prevaccination period to the postvaccination period, a phenomenon that has also been reported in other regions where PCV7 or PCV10 has been introduced (34), which is associated with an increase in the proportion of multidrug-resistant Spn19A (35). In this study, Spn19A decreased sensitivity changed from 0 to 66.7%. In Colombia, similar to other countries, this serotype has been associated with the ST320 clonal complex (36–38). PCVs decrease antimicrobial resistance to the extent that resistant and prevalent serotypes are included in them. Other factors, such as the nasopharyngeal carrying capacity and the inappropriate use of antibiotics, may be determining factors in the generation of resistance (39, 40). The introduction of vaccines with greater coverage in serotypes, especially against Spn19A, might help reduce resistance, along with the introduction of policies and strategies that promote an adequate and rational use of antibiotics (39, 40).

The increase in resistance affects the use of antibiotics, as observed for vancomycin in the transition and postvaccination periods, as its empiric use and its use in targeted treatment showed an important increase in the study population.

The use of dexamethasone as part of treatment has shown usefulness mainly in reducing sequelae and mortality associated with the disease (41, 42). However, the use of this drug by the study population was low.

Severe complications were observed in approximately half of the patients, and the high mortality rate, which has increased in recent periods, might be considered a consequence of the circulation of more virulent serotypes and greater resistance to antibiotics, although this finding was not statistically significant in this study (Table 2). These values are greater than those reported in other studies examining the burden of pneumococcal meningitis in children, where up to 67.3% of children were admitted to the intensive care unit, and only 23.1% required invasive mechanical ventilation (43). The severity seems to be greater with Spn19A infection in terms of complications and mortality, but the differences were not statistically significant.

Mass vaccination in Colombia had the expected effect, with an initial decrease in the number of cases related to the serotypes included in the vaccine and a subsequent increase that was probably secondary to the circulation of serotypes that are not included, especially Spn19A, with a significant effect on severity of the disease and resistance to antibiotics. These data have been confirmed by the laboratory reports of the National Institute of Health, which show variation in resistance patterns from an intermediate or resistant sensitivity of 28.3% for meningeal isolates to third-generation cephalosporins between 2006 and 2007 in children under 5 years of age to 35.3% for the period from 2017 to 2018 (13). Therefore, new strategies, including the use of vaccines with greater serotype coverage, strengthening of diagnostic strategies and awareness regarding the disease and improvements in therapeutic strategies using tools that reduce sequelae, complications and lethality, must be established in accordance with the objectives proposed by the WHO in the program “Defeating meningitis by 2030” (4, 44).

This study has the strength of being a multicenter study including a large number of patients with meningitis. To our knowledge, this study is the first to analyze the clinical behavior of acute bacterial meningitis after the introduction of PCV10 in Colombia. The limitations of this research include the type of data collected, which were retrospective in some cases, implying the lack of availability of some information. Another limitation is that the serotypes and vaccination status were not available for all patients, especially patients treated in the first period. During the third study period, molecular diagnostic techniques were introduced in seven hospitals of the network. This change complicates comparisons of the incidence of the disease with the rates in the first two periods. The incidence of the third period was also calculated excluding the six cases detected only with molecular tests to control for this limitation. The results of the study are related to the national data reported by the National Institute of Health of Colombia, which includes a representative sample and reduces the effects of these limitations.

ABM due to pneumococcus has high morbidity and mortality rates, and its care requires significant use of health resources. In the present study, reemergence of pneumococcal meningitis was observed partially due to the turnover in circulating serotypes after the implementation of PCV10, with an increase in the prevalence of serotypes that are not covered by PCV 10, especially Spn19A, and associated with greater antimicrobial resistance. These results are similar to findings from patients with other clinical presentations of IPD, such as bacteremic pneumonia and primary bacteriemia (22–24, 36, 45, 46), and to those reported by the national epidemiological surveillance system (3, 27–32). The data from this and other studies were analyzed by the National Council of Immunization Practices (Concejo Nacional de Prácticas de Inmunización-CNPI); in April 2022, the Ministry of Health and Social Protection of Colombia announced the change from PCV10 to PCV13, which has been administered since July 1, 2022, to children born since May 1, 2022. Continued surveillance is required to detect epidemiological changes after the inclusion of this vaccine.

## Data availability statement

The original contributions presented in the study are included in the article/Supplementary material, further inquiries can be directed to the corresponding author/s.

## Ethics statement

The studies involving human participants were reviewed and approved by HOMI-Fundación Hospital Pediátrico La Misericordia, Bogotá, Colombia. Clínica Universitaria Colombia-Clínica Pediátrica Colsanitas, Bogotá, Colombia. Fundación Cardioinfantil-Instituto de Cardiología, Bogotá, Colombia. Hospital Militar Central, Bogotá, Colombia. Unidad de Servicio de Salud Tunal, Bogotá, Colombia. Hospital Universitario Clínica San Rafael, Bogotá, Colombia. Unidad de Servicios de Salud Santa Clara, Subred Centro Oriente, Bogotá, Colombia. Los COBOS Medical Center, Bogotá, Colombia. Hospital Infantil Universitario de San José, Bogotá, Colombia. Fundación Valle de Lili, Cali, Colombia. Fundación Clínica Infantil Club Noel, Cali, Colombia. Centro Médico Imbanaco, Cali, Colombia. Hospital Universitario San Vicente Fundación, Medellín,Colombia. Hospital Pablo Tobón Uribe, Medellín, Colombia. Hospital Infantil Napoleón Franco Pareja, Cartagena, Colombia. Hospital Universitario del Valle, Cali, Colombia. Clínica Infantil Colsubsidio, Bogotá, Colombia. Written informed consent from the participants' legal guardian/next of kin was not required to participate in this study in accordance with the national legislation and the institutional requirements.

## Author contributions

Conception and study design, data collection, and writing of the final manuscript, editing, review, and approval: JF-A, GC-M, AL, JP, VM, WC, IG, SB, MÁ-O, CM, RB, JR, FE, CA-F, MS, MT, EL-M, PL, HP, NR, and AM. Statistical analysis: JF-A, GC-M, VM, and WC. Writing of the original manuscript: JF-A, GC-M, AL, JP, and WC. All authors contributed to the article and approved the submitted version.

## Funding

This work was funded through independent Grant Number WI235048 from the Colombian Association of Infectious Diseases (Asociación Colombiana de Infectología-ACIN)-central chapter to Pfizer SAS Laboratories. The authors are responsible for the information and its analysis. Pfizer Laboratories did not participate in the preparation or analysis of the data presented in this article. The institutional researchers did not receive any remuneration for their participation in the study.

## Conflict of interest

GC-M has received support from Pfizer, MSD (Merck Sharp and Dohme) and Sanofi Pasteur for participation in congresses and paid conferences, has participated in advisory boards and has received support from MSD for research. AL has received support from Pfizer and MSD (Merck Sharp and Dohme) for participation in congresses and paid conferences, has participated in advisory boards and has received support from MSD for research. JP has received support from Pfizer for participation in congresses. WC has received support from Pfizer, GSK and AstraZeneca for participation in congresses and paid conferences. IG has received support from Pfizer for participation in congresses and paid conferences. SB has received support from Pfizer for participation in congresses. MÁ-O has received support from Pfizer for participation in congresses. CM has received support from Pfizer for participation in congresses. RB has received support from Pfizer and MSD for participation in congresses. FE has received support from MSD for research. JR has received support from Pfizer for participation in congresses and paid conferences. EL-M has received research support from MSD, GSK, Sanofi Pasteur, Pfizer, Takeda and Janssen. PL has received research support from GSK, Sanofi Pasteur and Takeda. The remaining authors declare that the research was conducted in the absence of any commercial or financial relationships that could be construed as a potential conflict of interest.

## Publisher's note

All claims expressed in this article are solely those of the authors and do not necessarily represent those of their affiliated organizations, or those of the publisher, the editors and the reviewers. Any product that may be evaluated in this article, or claim that may be made by its manufacturer, is not guaranteed or endorsed by the publisher.
