# Supplementary material for: Changes in the incidence of acute bacterial meningitis caused by Streptococcus pneumoniae and the implications of serotype replacement in children in Colombia after mass vaccination with PCV10
Source: Front Pediatr. 2022 Sep 23;10:1006887. doi: 10.3389/fped.2022.1006887 (PMC9545348; doi:10.3389/fped.2022.1006887)
Supplement: Supplementary file 1 [file Table_1.pdf]

TABLE S1 **Results of cerebrospinal fluid analysis**

Variable	Result	Range
**Glucose mg/dl (median, IQR)**	10 (1.6 - 39.5)	0 - 85
Glycorrhachia/serum glucose ratio (median, IQR)	0.05 (0.01 - 0.29)	0.0 - 0.99
**Proteins g/dl (median, IQR)**	272.1 (115.2 - 335.4)	13.3 - 2127
**Red blood cells /mm3 (median, IQR)**	76 (15 - 838)	0 - 135000
**Leukocytes /mm3 (median, IQR)**	220 (36 - 1250)	0 - 15100
Polymorphonuclear /mm3 (median, IQR)	115.5 (32.8 - 1036.8)	0 - 14647
Mononuclear /mm3 (median, IQR)	20 (1.8 - 149.8)	0 - 1850
PMN predominance (%)	50 (61.7%)	-
No data (%)	25 (30.9%)	-
**Gram staining**		
Gram-positive cocci	57 (70.4%)	-
Negative Gram staining	17 (21%)	-

PMN: Polymorphonuclear
