# Supplementary figures and images for: Changes in the incidence of acute bacterial meningitis caused by Streptococcus pneumoniae and the implications of serotype replacement in children in Colombia after mass vaccination with PCV10
Source: Front Pediatr. 2022 Sep 23;10:1006887. doi: 10.3389/fped.2022.1006887 (PMC9545348; doi:10.3389/fped.2022.1006887)

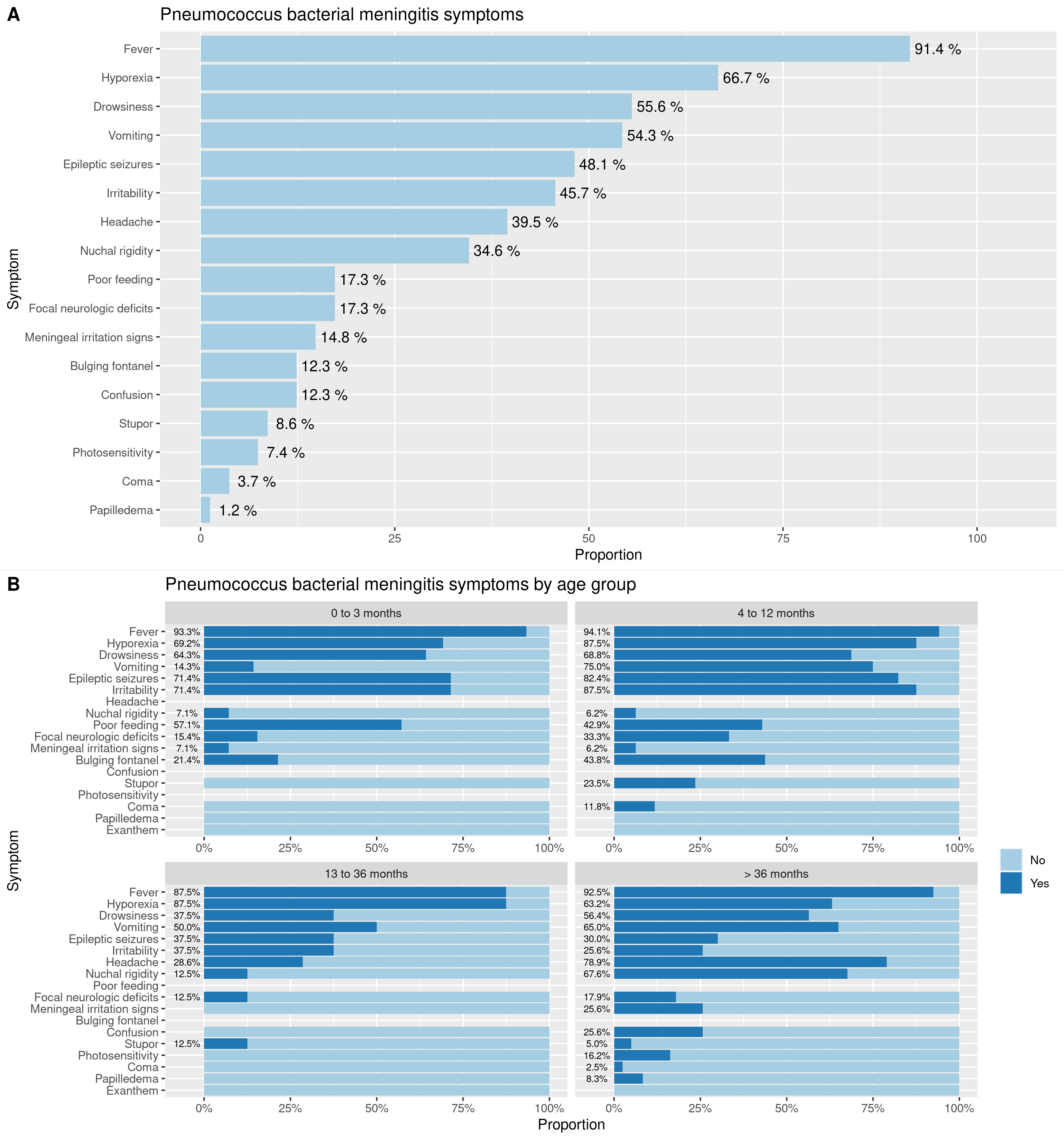

Supplement: Supplementary file 2 [file Image_1.JPEG]

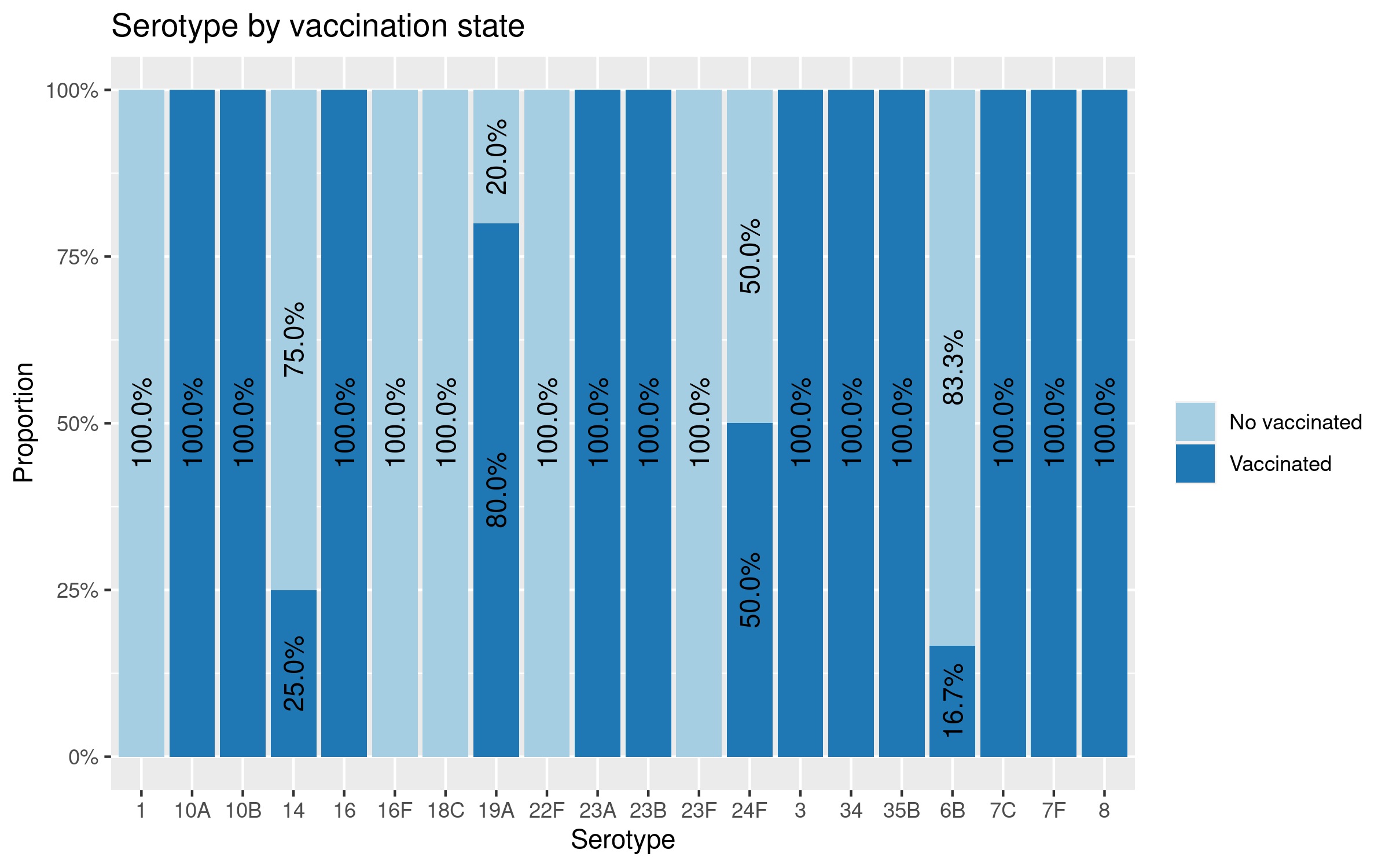

Supplement: Supplementary file 3 [file Image_2.JPEG]
